# Mapping the effect of antimicrobial resistance in poultry production in Senegal: an integrated system dynamics and network analysis approach

**DOI:** 10.3389/fvets.2023.1189109

**Published:** 2023-07-13

**Authors:** Joshua Aboah, Babacar Ngom, Eves Emes, Awa Gueye Fall, Moutar Seydi, Ardiouma Faye, Michel Dione

**Affiliations:** ^1^International Livestock Research Institute (Senegal), Saint-Louis, Senegal; ^2^Commonwealth Scientific and Industrial Research Organisation, St Lucia, QLD, Australia; ^3^Directorate of Veterinary Services, Ministry of Livestock and Animal Productions, Dakar, Senegal; ^4^Vaccine Centre, London School of Hygiene and Tropical Medicine, London, United Kingdom; ^5^Université Cheikh Anta Diop de Dakar, Département de Sociologie, Dakar, Senegal

**Keywords:** antibiotics, systems thinking, poultry, antimicrobial, livestock systems

## Abstract

The impact of antimicrobial resistance (AMR) extends beyond the farm-level to other stakeholders warranting the need for a collaborative approach to combat AMR while optimising production objectives and safeguarding human health. This study maps out the effect of AMR originating from poultry production in Senegal and highlights the entry points for interventions from stakeholders’ perspectives. A causal loop diagram (CLD) was developed following a group model building procedure with 20 stakeholders and integrated with network analysis by translating the CLD into an unweighted directed network. Results indicate that with an eigenvector centrality of 1, 0.85, and 0.74, the production cost, on-farm profit, and on-farm productivity, respectively are the most ranked influential variables driving the complexity of AMR in the poultry production system. Two reinforcing feedback loops highlight the dual benefits of improving on-farm productivity and increasing on-farm profit. However, one balancing feedback loop that revolves around the causal link between producers’ investment in qualified human resource personnel to ensure good farm management practices underline the financial implication of producers’ investment decisions. The findings provide precursory groundings for the development of a quantitative SD model, the formulation of intervention scenarios and *ex-ante* impact assessment of the cost-effectiveness of the interventions.

## 1. Introduction

Intensification of livestock production has led to increased use of veterinary pharmaceutical agents including antimicrobials and antibacterial. Extant studies have indicated that antimicrobial resistance (AMR) of livestock origin is high in low-and middle-income countries (LMICs) like Uganda and Malawi (1, 2), especially in poultry and pigs (3, 4). From 2000 to 2018, the proportion of bacteria showing AMR increased from 0.15 to 0.41 in chickens globally (5). One of the major drivers of AMR is the misuse of antimicrobials, which is posited to be more pronounced in low-income countries where veterinary services are limited, poor animal husbandry is practised and access to antimicrobials is poorly controlled (6–8). With increasing antimicrobial usage in animal production (9), and human pathogens being zoonotic, there is a pending danger of AMR build-up in humans, which can lead to clinical failure causing increases in treatment costs and sometimes death (10).

Biosecurity measures are one of the espoused ways to curb AMR in poultry production. For instance, the spacing of birds and proper disposal of manure and farm waste have been noted to reduce *Salmonella* in poultry while high bird density increases *Salmonella* which may require antibiotics to treat infections (6). Good biosecurity measures should lead to high productivity but if the practices are expensive, then there is a countervailing effect on the benefits. Thus, there is a need to strike a balance of simultaneously reducing AMR and increasing profit (11). The situation is exacerbated when animal production occurs in peri-urban settings where there are relatively high human-animal interactions via informal livestock trade, increasing the exposure of humans to the risk of transmission of zoonotic diseases.

According to the World Health Organization (WHO), AMR is one of the ten global health challenges that leads to treatment failure and causes mortality of infected animals or humans. Livestock production is affected by resistant bacteria, and the impact extends beyond the farm-level to consumers in the value chain (12, 13). However, there is limited emphasis on antimicrobial use in animal production in developing countries (14). Moreover, there are conflicting objectives for the different stakeholders in the livestock value chain. While poultry farmers increase antimicrobial use as a cost-saving mechanism against disease, consumers’ demand for poultry products that are not contaminated with residues of the antimicrobials is also increasing. Hence, there is a need for a collaborative effort and a holistic approach to combat AMR (2), while optimising production objectives, and safeguarding human health. This effort requires a multidisciplinary approach that enables inter-sectoral dialogues among relevant stakeholders. System dynamics (SD) modelling facilitates a participatory model building process that allows stakeholders to co-conceptualise the model that highlights the various interactions that produce systemic problem-of-interest, in this case, AMR.

Although poultry and pigs have the highest levels of antimicrobial resistance (AMR) among livestock in the literature (3, 4), this study focused on poultry due to its importance for food security in Senegal. Therefore, this study seeks to map out the effect of AMR in peri-urban poultry production systems in Senegal and highlight the entry points of interventions that simultaneously maximise profit and minimise the economic loss for producers. The findings serve as a preliminary step for the development of a quantitative SD model that can be used for *ex-ante* impact assessment of AMR.

## 2. Materials and methods

System dynamics (SD) modelling has been used extensively for *ex-ante* assessment of different livestock systems (15–17). The approach allows for qualitative and quantitative assessment of complexity in agricultural value chains using causal loop diagrams (CLDs) and stock and flows diagrams (SFDs), respectively. CLDs help to capture the different variable interactions that shape the system’s structure and map out the feedback loops in complex systems. However, analyses based on only CLDs are critiqued as inconclusive. Hence, complex system analysis using CLDs is augmented by the integration of network analysis (18). This integrated approach has been applied in the study of childhood obesity (19) and community-based obesity (20).

### 2.1. The integrated approach: participatory SD modelling

**Figure 1:**
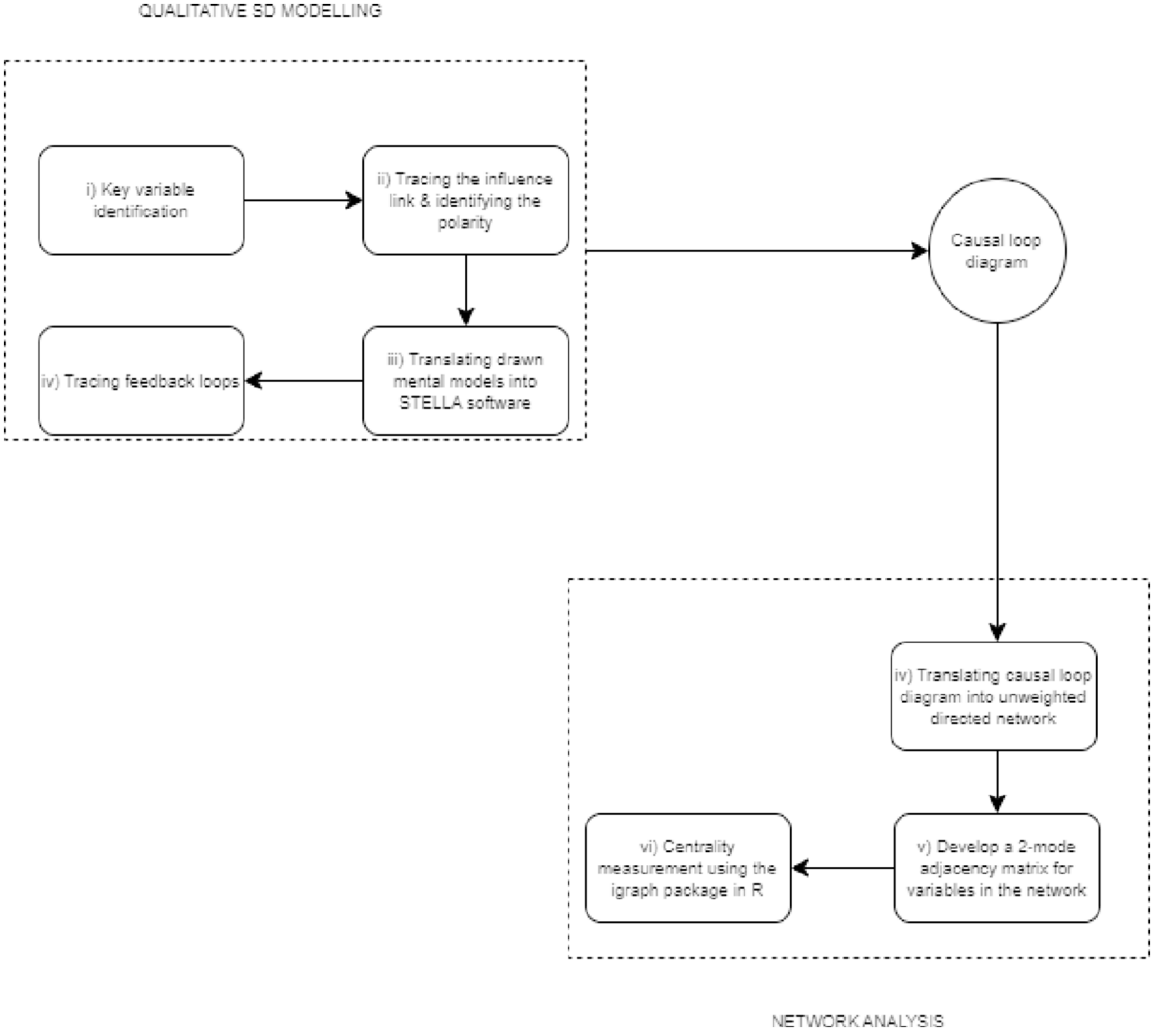
The steps involved in the integrated analytical approach.

In this study, the integrated qualitative system dynamics (SD) modelling and network analysis was adopted to map out the effect of AMR in the peri-urban poultry production system in Senegal and highlight the key entry points of interventions that simultaneously maximise profit for poultry producers and minimise the economic loss for producers. Figure 1 is an illustration of the processes involved in the integrated analytical approach.

For the qualitative SD modelling component of the integrated approach, a participatory group model building procedure was used to develop a causal loop diagram (CLD). The first two participatory steps (steps i and ii) in Figure 1 were performed during a two-day stakeholders’ workshop organised by the International Livestock Research Institute (ILRI) in Senegal, and 20 stakeholders from different sectors including veterinary officers, policymakers, poultry farmers, university researchers, and input dealers. The stakeholders were selected purposively based on their expertise and roles in the poultry value chain. The selected stakeholders represented different parts of the poultry value chain and their views on the issues discussed reflected their interests, which were not always congruent with the views held by other stakeholders. The stakeholders received official invitations to the workshop via email, which included detailed information about the agenda (see Appendix A). Stakeholders’ consent was obtained through their agreement to participate in the workshop and sign the attendance sheet. Table 1 presents the stakeholders’ profiles. This study involving human participants received ethical approval from the International Livestock Research Institute (ILRI) Institutional Research Ethics Committee under reference number ILRI-IREC2022-44.

**Table 1:** Profile of participants involved in the group model building.

Number of participants	Type of organization	Role in the poultry value chain
1	National Livestock Research	Lead research in livestock production and health at the national level.
2	International Livestock Research Institute	Implement research in livestock and One Health at the regional and international levels
3	Ministry of Livestock/Government	Develop policies and regulations at the national level to guide investment and regulate veterinary drugs quality and use in the country
3	Academia	Conduct training of veterinarians and biologists to earn degrees in animal and public health
3	Veterinary/Private	Conduct treatment and monitoring of diseases on farms and advise producers on how to improve the health of their herds
2	Poultry Producers/Small and Medium scale	These categories of actors are critical if one wants to instigate change in the poultry production systems. Producers purchase veterinary drugs, call upon vets for treatments. They have a strong decision-making power towards their poultry farms.
1	Veterinary Drug Importer	Oversee importation and distribution of veterinary drugs through retailers
1	Human Laboratory	Conduct AMR surveillance on patients at the hospital
1	Agricultural Policy and Strategic Research	These actors are independent and belong to non-profit organizations carrying strategic research and studies in agriculture and policy nationally and to some extent at the regional level to provide useful recommendations to policy makers
1	Veterinary Association	In charge of regulating the profession and providing support to veterinarians especially for those who wish to establish private business
2	One Health—Intersectoral	Coordinate and support good implementation of intersectoral activities in AMR including research and development projects, as well as capacity building
1	Laboratory Research—Microbiology	Research laboratories in biology and human health
1	Poultry Industry/Large Scale	These actors are the industrial poultry sector holding the major share in the poultry and egg market in the country

**Figure 2:**
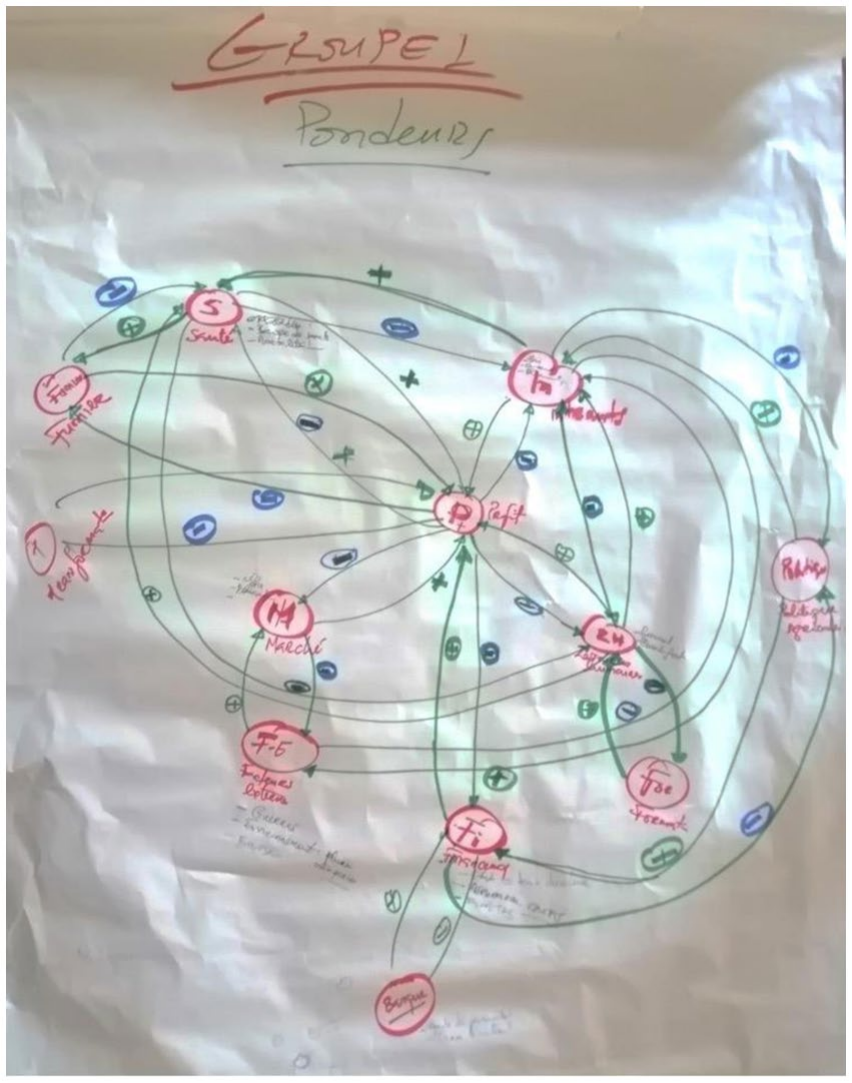
A drawn causal loop diagram by stakeholders.

For the participatory steps, participants were grouped into two poultry production systems—layers and broilers to elicit divergent views for the variable mapping. Producers were assigned to their groups based on the production system they are engaged in. The remaining stakeholders were randomly assigned to the layers and broilers group while ensuring that participants from the same institution were not in the same group. This grouping allowed for a more nuanced and comprehensive understanding of the variable interactions within each system. However, the ultimate aim was to converge these views into a consolidated explanation, as reflected in the causal loop diagram. The participatory sessions were facilitated by two researchers (an epidemiologist and a system dynamics modeller). After initial discussions among stakeholders, two output variables (i.e., on-farm profit and antimicrobial use) were selected as the focal outputs. Using on-farm profit as the focal objective, participants were asked to (i) identify the primary influencing variables (*P*_1_) that cause changes in on-farm profit (Profit _[farm]_), (ii) draw a connecting arrow from *P*_1_ to Profit _[farm],_ and (iii) indicate the polarity of the linkage. Supposing an increase (or decrease) in *P*_1_ causes Profit _[farm]_ to increase (or decrease), then a positive polarity was specified. If an increase in *P*_1_ causes a decrease in Profit _[farm]_ then a negative polarity was specified. The procedure was repeated for the link between secondary influencing variables (*S*_1_) and *P*_1_, and the link between tertiary influencing variables (*T*_1_) and *S*_1_. Secondary influencing variables are preceded and succeeded by primary and tertiary influencing variables, respectively. Likewise, presenting antimicrobial use as the focal objective, participants mapped out the CLD for antimicrobial use following the procedure described for on-farm profit. The process allowed stakeholders to interact and debate among themselves about singular causal relationships between two variables at a time. In these iterative deliberations, the stakeholders offered their opinions on a singular causal relationship based on their experiential learning and use the opinions of other stakeholders as sounding boards to reach a consensus. Figure 2 shows the CLD developed from the participatory group model building process.

After mapping the effects separately for on-farm profit and antimicrobial use at the farm-level, participants were asked to combine the two CLDs by identifying common variables in both CLDs. Also, participants used colour stickers to identify and rank the pathways of interventions to increase on-farm profit and reduce AMR. The drawn CLDs were translated to a soft copy with the aid of the STELLA Architect software. These steps were completed by the modeller in the research team.

### 2.2. The integrated approach: network analysis

The developed CLD was translated into an unweighted directed network to support the assessment of the centrality of key variables in the CLD as entry points of intervention pathways. The relationship in the directed network was modelled as a graph consisting of nodes (variables in the CLD) and causal links (influence). A two-mode adjacency matrix was generated from the directed network to facilitate the estimation of centrality measures. The adjacency matrix was constructed by denoting a link from variable ( $a_{i}$ ) to variable ( $a_{j}$ ) as 1, and 0 was assigned when there is no link between the two variables. Two centrality measures—eigenvalue centrality and out-degree centrality (*C*_d_) were estimated using the igraph package in R (21). In the context of CLDs, out-degree centrality (*C*_d_) is the number of links (influence) that a variable has. Therefore, a variable that has more links or influences more variables in the CLD has the highest out-degree centrality score. C_d_ is estimated as in Eq. 1 (22).

(1) $$C_{d}=\underset{i}{\sum }a_{ij}\;$$

The eigenvector centrality is the estimated score for a variable ( $a_{i}$ ) that has links with (or influences changes in) the variable with the highest *C*_d_. *λ* is the constant (eigen value), $a_{ij}$ is a link from node*_i_* to node*_j_*, and *e_i_* is the vector of centrality of *e* = (*e*_1_, *e*_2_, …). Eigenvector centrality (*e_j_*) was estimated as shown in Eq. 2 (23).

(2) $$e_{j}=\lambda^{-1}\underset{j}{\sum }a_{ij}e_{i}$$

## 3. Results

### 3.1. Mapping the variable interaction revolving around on-farm profit

**Figure 3:**
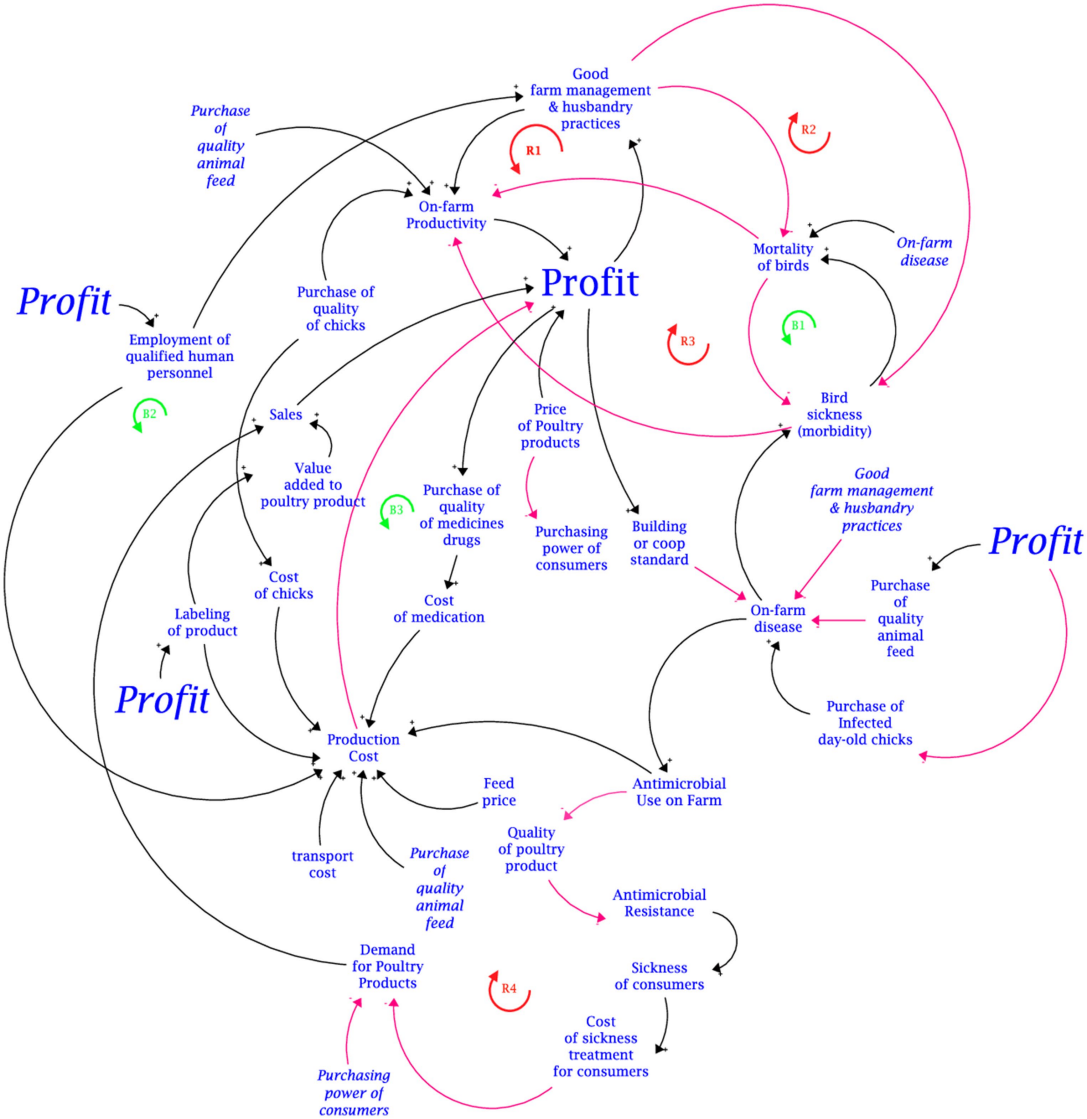
Causal loop diagram on the impact of antimicrobial use of farm-level profit.

**Figure 4:**
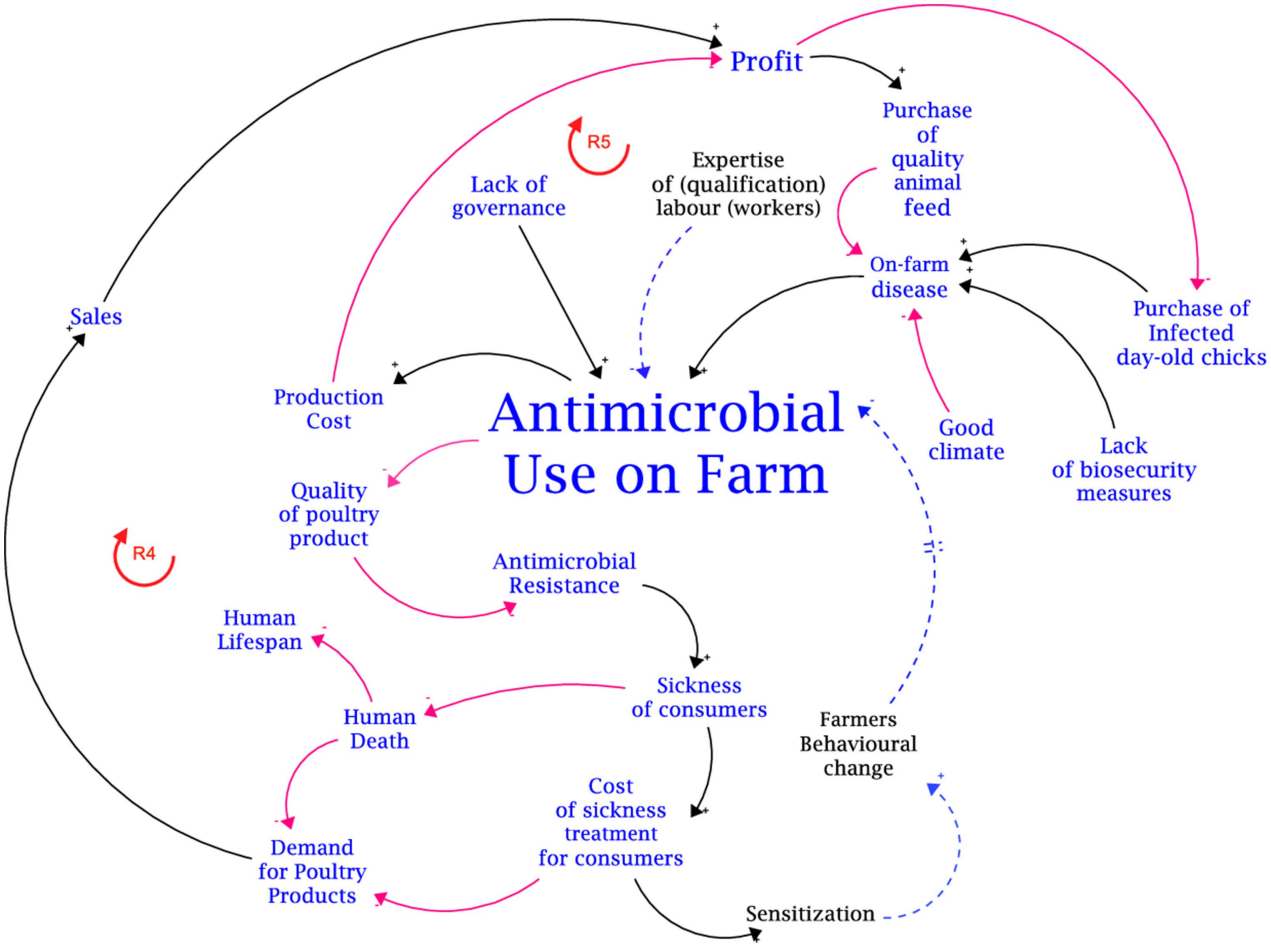
Causal loop diagram from disaggregated for only antimicrobial use on farm.

The disaggregated translations of the mapped-out causal loop diagram (CLD) developed from the group model building with stakeholders for the two focal output variables are presented in Figures 3, 4. These figures represent the consolidation of the different causal loop diagrams drawn by the two groups (i.e., broilers and layers group). In essence, there was no significant difference in the variable interaction between the two causal loop diagrams drawn by the groups. The aggregated CLD is presented in Appendix B. The links between the variables are distinguished by colour. Red links are negative causal links indicated by a negative (−) polarity. The black links are positive causal links indicated by a positive (+) polarity. The blue links are the identified pathways for interventions. The CLD captures the interactions of variables revolving around two main outcomes: on-farm profit and antimicrobial use on farms. The primary influencing variables identified to have negative causal links with on-farm profit can be aggregated as the production cost. Thus, the negative causal link between total production cost and on-farm profit is exacerbated by the positive causal link between production cost and these secondary influencing variables: feed price, cost of day-old chicks, value addition (in terms of labelling of products), and the cost of medication and antimicrobial use.

The primary influencing variables with positive causal links to on-farm profit are on-farm productivity levels, sales volume (eggs and meat), and the price of poultry products (meat and eggs). For the layer and broiler groups, on-farm productivity is determined as the number of eggs produced and kilograms of birds, respectively. On-farm productivity is positively influenced by the adoption of good farm management and husbandry practices (like biosecurity measures and vaccination), the quality of chicks purchased, and the quality of feed given to the birds. A reinforcing feedback loop (R1) was traced along the positive causal links from; on-farm profit to good farm management practice; good farm management practice to on-farm productivity; and on-farm productivity to on-farm profit. Another reinforcing feedback loop (R2) was traced around the moderating effect of the negative causal link between good farm management practice and morbidity (due to the sickness of birds) and the negative causal link between morbidity and on-farm profit. Morbidity and mortality of the birds due to diseases have a negative causal influence on on-farm productivity. Concerning on-farm profit, the morbidity of sick birds has a moderating effect on the negative causal link between on-farm profit and bird mortality via its positive causal link with mortality. Stakeholders averred that an increase in bird mortality causes the number of sick birds to reduce. Thus, a balancing feedback loop (B1) is created.

Positive causal links with on-farm profit were mapped for these variables: the price of poultry products (meat and eggs), quality of chicks, sales (meat and eggs), and good farm management practices. The positive causal relationship between sales and on-farm profit serves as a mediating causal link between the demand for poultry products. Thus, *cetris paribus*, higher demand translates to higher sales and ultimately higher profit assuming demand is inelastic. Stakeholders posited that the employment of qualified human resource personnel presents two contrasting effects that highlight the potential need for trade-off analysis. On one hand, it leads to two desirable reinforcing feedback loops (R1 and R2). From another point of view, the employment of qualified human resource personnel increases production costs and decreases profit, creating a balancing feedback loop (B2). Thus, the trade-off is highlighted by the moderating effect of the employment of qualified human resources and the positive causal link between the adoption of good farm management practice and on-farm profit, and the negative causal link between the employment of qualified human resources and on-farm profit.

### 3.2. Mapping the variable interaction revolving around antimicrobial use

The second focal variable is antimicrobial use (AMU) on the farm. The primary factors of AMU on farms are the lack of governance of the value chain for antimicrobial uses, the incidence of disease on the farm, and bird mortality. All these factors have positive causal links with AMU on the farm-level. Increased AMU reduces the quality of poultry products in terms of residue build-up, which in turn, causes an increase in antimicrobial resistance (AMR) in consumers of poultry products leading to an increased incidence of sickness in consumers and treatment costs. However, it can be safely assumed that consumers will rationally react to the effect on AMR by decreasing demand for poultry products. The decrease in demand will affect sales and ultimately the on-farm profit for producers.

The financial implication of AMU on the farm is highlighted by the moderating effect of antimicrobial use on the relationship between total production cost and on-farm profit. Thus, as AMU increases production cost increases and the potential on-farm profit decreases. This moderating effect can be attributed to the balancing feedback loop (B3) which is traced by the positive causal link from on-farm profit to the purchase of quality medicines and pharmaceutical agents for poultry production and production cost, and the negative causal link from production cost to on-farm profit. The balancing feedback loop suggests that the availability and accessibility of cheap antimicrobials will encourage continuous usage due to the low economic effect (decrease in on-farm profit). Conversely, a high cost of antimicrobials can either be a demotivation for continuous usage or instigate producers to resort to falsified and substandard antimicrobials that can stimulate AMR.

### 3.3. Rankings of interventions

**Figure 5:**
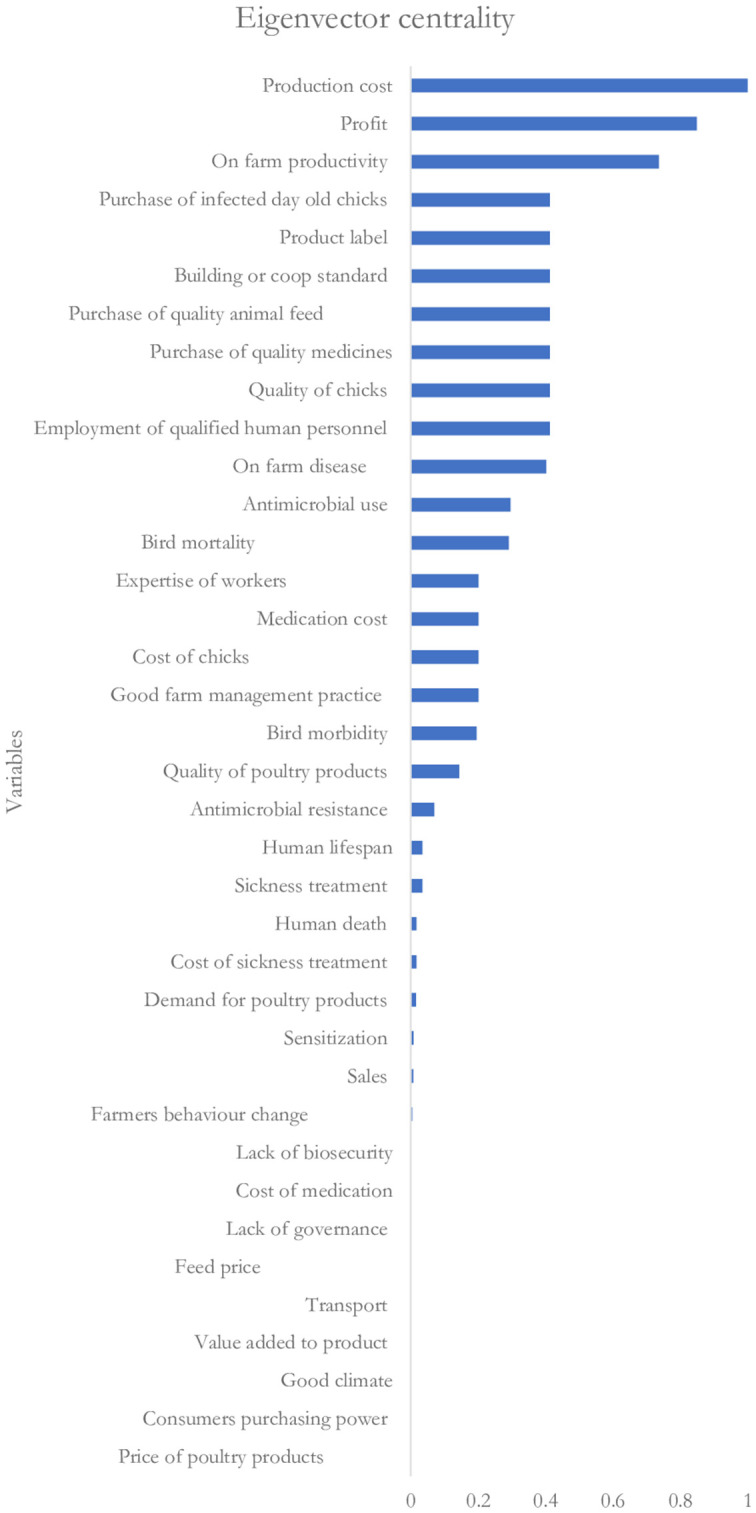
Ranking based on the eigenvector centrality score.

**Figure 6:**
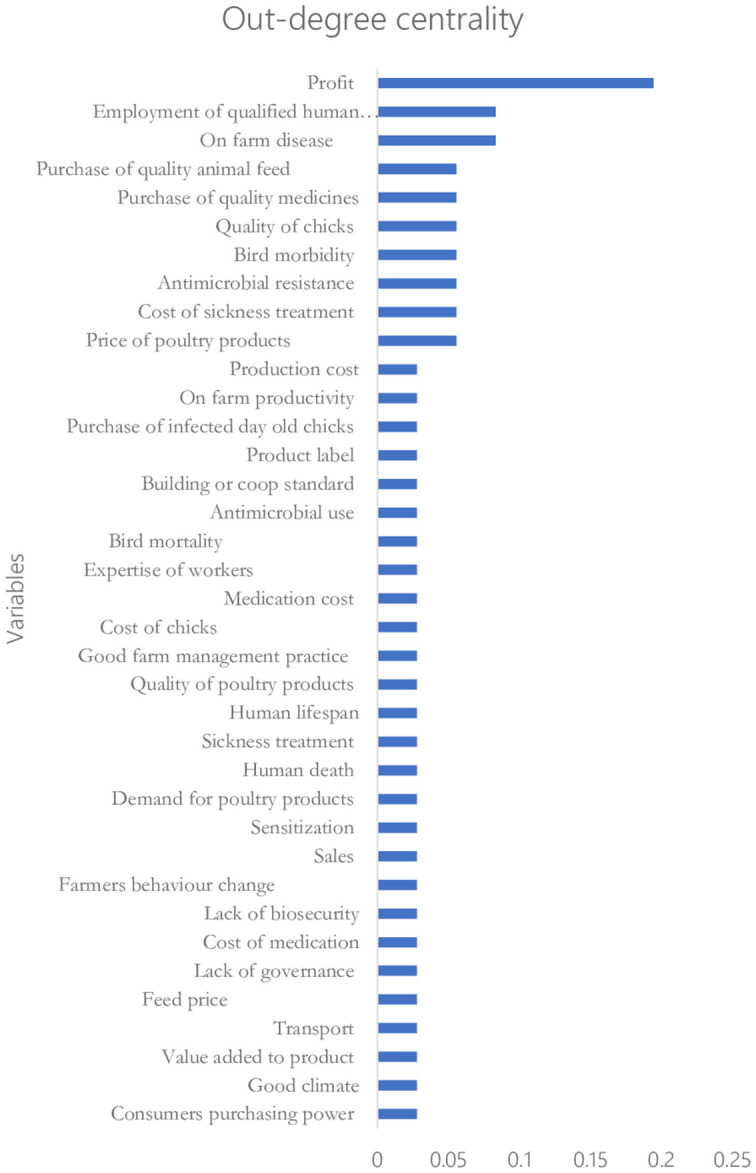
Ranking based on the out-degree centrality Score.

Figures 5, 6 show the rankings of the variables in the CLD based on the eigenvector centrality and out-degree centrality measures, respectively. Results on the ranking based on the eigenvector centrality indicate that the production cost (with a score of 1) influences the key variable that affects changes in most of the variables in the CLD. This is followed by on-farm profit and on-farm productivity with eigenvector centrality scores of 0.85 and 0.74, respectively. Also, the results in Figure 6 indicate that the on-farm profit is the highest-ranked influential variable (with an out-degree centrality score of 0.2) followed by on-farm disease and producers’ decision to employ qualified human resource personnel with the out-degree centrality scores of 0.1 each.

The variable rankings showed concordance with the proposed interventions by stakeholders presented in Table 2. Generally, the proposed interventions are preventive in nature and mostly focused on the farm-level. Hence, the proposed interventions are viewed as ways of controlling AMR and do not necessarily address the ranked variables in the network analysis. However, these interventions have financial implications on the production cost and focus on reducing the incidence of disease occurring at the farm-level. One group ranked governance of value chain activities in relation to antimicrobial use as the highest-ranked intervention. This intervention, which is crucial in LMICs like Senegal, will serve as a regulatory measure to guide producers in the procurement and use of antimicrobials for poultry production.

**Table 2:** Ranking of proposed interventions to curb AMR in poultry production.

Proposed interventions	Group 1 (layers) rank	Group 2 (broilers) rank
Governance of value chains to regulate antimicrobial use	1st	Not ranked
Employing qualified employees on farm	2nd	Not ranked
Improving farm environment to reduce disease incidence	3rd	5th
Improving biosecurity measures at farm level	4th	Not ranked
Procurement of healthy day-old chicks	5th	1st
Procurement of good feed	6th	3rd
Good housing	Not ranked	2nd
Improving medication of birds	Not ranked	4th

## 4. Discussion

The drivers of AMR are complex, multi-faceted and multi-sectoral (2, 8). However, different studies on AMR conducted in India, Brazil, China, and Malawi have noted that the high use of antimicrobials, which is often intended to promote growth in animals, is the primary driver of AMR (14, 24). The lack of regulations and AMR surveillance systems increases the likelihood of overuse of antimicrobials in small-scale intensive production (2). In corroboration with the observations made in a different study (25), this study’s findings on the moderating effect of antimicrobial use on the relationship between production cost and on-farm profit indicate that the overuse of antimicrobials as a driver of AMR is an economic decision taken by producers to achieve production efficiency, improve yield and profit. Consequently, the motive of reducing production cost is a root cause for overuse of antimicrobials.

Two strategies accounting for the overuse of antimicrobials can be attributed to the economic motive of minimising production cost. These strategies are inferred from the relationship between on-farm profit and the adoption of good farm management and husbandry practices, and the relationship between production cost and on-farm profit in the causal loop diagram (Figure 3). The first strategy is the use of cheaper substandard antimicrobials. Although in rare circumstances the use of substandard antimicrobials is due to a lack of market access, often in peri-urban areas market access is not an issue. Hence, substandard antimicrobials are synonymous with cheap antimicrobials. Indeed, it is noted that in Malawi, the cost of antibiotics influences its use (2). However, there is a neglect of substandard and falsified antibiotics as a contributing factor of AMR, and a higher prevalence of substandard and falsified antibiotics is reported in LMICs (26). Hence, there is a strong likelihood for producers to resort to cheaper substandard antibiotics when the recommended antibiotics are expensive. This phenomenon can be linked to the political aspects of AMR drivers (i.e., lack of regulatory bodies) (8).

The second strategy is the overuse of antimicrobials as a cheaper alternative for recommended biosecurity measures for animal production. Biosecurity measures are noted examples of preventive measures to reduce antibiotics usage in poultry production. Yet, the financial implication of these measures needs to be critically examined to ascertain the return on investment for producers who will normally make economically sound production decisions without necessarily considering the ramifications post-farmgate. Thus, in place of implementing biosecurity measures, producers will overuse antimicrobials in poultry as a cheaper precautionary measure compared to ensuring good husbandry conditions like cleaning pens in intensive production systems (2).

The complexity required to deal with the problem of AMR is apparent and the need for collaborative participation of stakeholders as a principal solution for AMR is recommended (10). Also, aside from the overuse of antibiotics as a primary driver, there are other factors that contribute to increased prevalence (14). Therefore, there is a need to understand other factors aside from the consumption of antimicrobials as a major driver of resistance evolution. These results could serve as strong recommendations to strengthen the cross-sector collaboration approach to tackle farm-level AMR. The national AMR control Strategy for Senegal promotes this approach to reach its objectives of reducing AMR in humans, animals, and the environment.

## Conclusion

In this study, a participatory system dynamics modelling approach was adopted to map out the effect of antimicrobial resistance (AMR) on on-farm profit in poultry production in peri-urban areas in Senegal. One of the main findings of the paper is that the primary influencing drivers of AMR are production cost and on-farm profit. Thus, the drivers are economic in nature, and the economic motive for overuse of antimicrobials is to reduce production cost. Also, this paper shows that the moderating effect of antimicrobial use on the relationship between total production cost and on-farm profit suggests that the availability and accessibility of cheap and substandard antimicrobials will encourage continuous usage (particularly when there is a lack of regulation) due to the low economic effect on on-farm profit. However, there are unintended consequences that need to be considered.

Conversely, a high cost of antimicrobials can be demotivation for continuous usage or instigate producers to resort to quack and substandard versions of antimicrobials that can stimulate antimicrobial resistance. Thus, there is a need for a regulatory framework to streamline and monitor the use of antimicrobials in animal production.

The adoption of a participatory approach gave stakeholders a platform to dialogue and co-create solutions for AMR. A system thinking method facilitated a holistic understanding and appreciation of AMR by highlighting the complexity and multi-faceted nature of AMR. Also, the approach allowed stakeholders to conceptualise the unintended consequences of the decisions beyond their immediate (focal) impact. Also, although the CLD developed from the qualitative SD modelling, a participatory approach limits the subjectivity in the CLD developed when different stakeholders engage to exchange opinions and map the causal relationships and interactions in the model.

### Limitations

This study adopted a participatory approach to elicit stakeholders’ views on antimicrobial use at the farm level. Therefore, the findings are valuable within the specific context of Senegal and may not be generalisable to other developing countries. Consequently, the findings may not be relevant in other geographical contexts. Additionally, the assumption of an equal weight of influence for all the variables in the network analysis ignores the concept of leverage points whereby changes in different variables do not necessarily cause the same level of change in other variables. Thus, although the integration of network analysis with qualitative system dynamics modelling abates the criticism of the inconclusiveness of results generated from qualitative SD modelling approaches, there is a need to quantitatively assess *ex-ante* the impact of the key interventions highlighted in this study. Thus, this study’s findings provide a precursory grounding for the cost effectiveness assessments of different scenarios of interventions to curb AMR.

## Data availability statement

The original contributions presented in the study are included in the article/Supplementary material, further inquiries can be directed to the corresponding author.

## Ethics statement

Stakeholders’ consent was obtained through their agreement to participate in the workshop and sign the attendance sheet. This study involving human participants received ethical approval from the International Livestock Research Institute (ILRI) Institutional Research Ethics Committee under reference number ILRI-IREC2022-44.

## Author contributions

MD and JA: conceptualization and validation. JA: methodology, software, formal analysis, writing—original draft preparation, and visualisation. MD, JA, EE, BN, AGF, MS, and AF: investigation and resources. JA, EE, and MD: writing—review and editing. MD: supervision, project administration, and funding acquisition. All authors contributed to the article and approved the submitted version.

## Funding

This work was funded as part of the JPIAMR consortium SEFASI with funding from SIDA grant number APH002001.

## Conflict of interest

The authors declare that the research was conducted in the absence of any commercial or financial relationships that could be construed as a potential conflict of interest.

## Publisher’s note

All claims expressed in this article are solely those of the authors and do not necessarily represent those of their affiliated organizations, or those of the publisher, the editors and the reviewers. Any product that may be evaluated in this article, or claim that may be made by its manufacturer, is not guaranteed or endorsed by the publisher.
