# Supplementary material for: Mapping the effect of antimicrobial resistance in poultry production in Senegal: an integrated system dynamics and network analysis approach
Source: Front Vet Sci. 2023 Jul 13;10:1189109. doi: 10.3389/fvets.2023.1189109 (PMC10374361; doi:10.3389/fvets.2023.1189109)
Supplement: Supplementary file 1 [file Data_Sheet_1.pdf]

Appendix A Agenda for the stakeholders' workshop

Schedule	Activities	Responsible
Day 1 (26/9/2022)		
8h30-9h00	Message from the Representative of the Minister of Livestock and Animal Production<br>Message from the President of the Order of Veterinary Doctors<br>Message from the representative of the Permanent Secretary of the High National Council for Global Health Security and One Health	International Livestock Research Institute (ILRI)
09h00-09h15	Presentation of the SEFASI project	ILRI
09h15-09h40	Presentation of the results of the survey knowledge, Attitudes and Practices in Poultry farms in Dakar and Thiès	- London School of Hygiene & Tropical Medicine (LSTMH)<br>- Inter-State School of Sciences and Veterinary Medicine (EISMV)
09h40-10h00	Discussions and Implications of the results	Directorate of Veterinary Services (DSV)
**10am-10.30am**	**Coffee break**	
10h30-11h00	Systematic review of the literature on the prevalence of *Salmonella* sp . And *Escherichia* coli and antibiotic resistance profiles in poultry and humans in Senegal.	Pasteur Institute of Dakar (IP)
11h00-11h30	Presentation of the AHMME model for the evaluation of the impacts of the RAM on the One Health interface	LSTMH
11h30-12h00	Presentation of the *panel regression model* for the evaluation of the impacts of RAM at the One Health interface	LSTMH
12h00-12h30	Presentation of the "System Dynamic Model" (SDM) for the evaluation of the impacts of RAM on the farm	ILRI
12:30-1:00 p.m.	Discussions on models (strengths, weaknesses, opportunities)	LNERV
**1:00 p.m. -2:00 p.m.**	**lunch break**	
2:00 p.m.-4:00 p.m.	SDM - session 1: Map the impact of production decisions on antimicrobial use (with stakeholders)	ILRI
**4:00 p.m.-4:30 p.m.**	**Coffee break**	
4:30-5:00 p.m.	Summary of the day and end	EISMV
Day 2 (09/27/2022)		
09h00-11h00	SDM - session 2: Intervention planning (with actors)	ILRI
**11h00-11h30**	**Coffee break**	
11:30 a.m.-1:00 p.m.	SDM - session 3: Parameterization of variables	ILRI
**1:00 p.m.-2:00 p.m.**	**lunch break**	
2:00 p.m.-2:30 p.m.	Roles and responsibilities of each partner and how to materialize the partnership	ILRI
2:30-3:00 p.m.	Discussions the formation of the SEFASI Knowledge Hub	ILRI
3:00-3:30 p.m.	Conclusions	

Appendix B Aggregated causal loop diagram

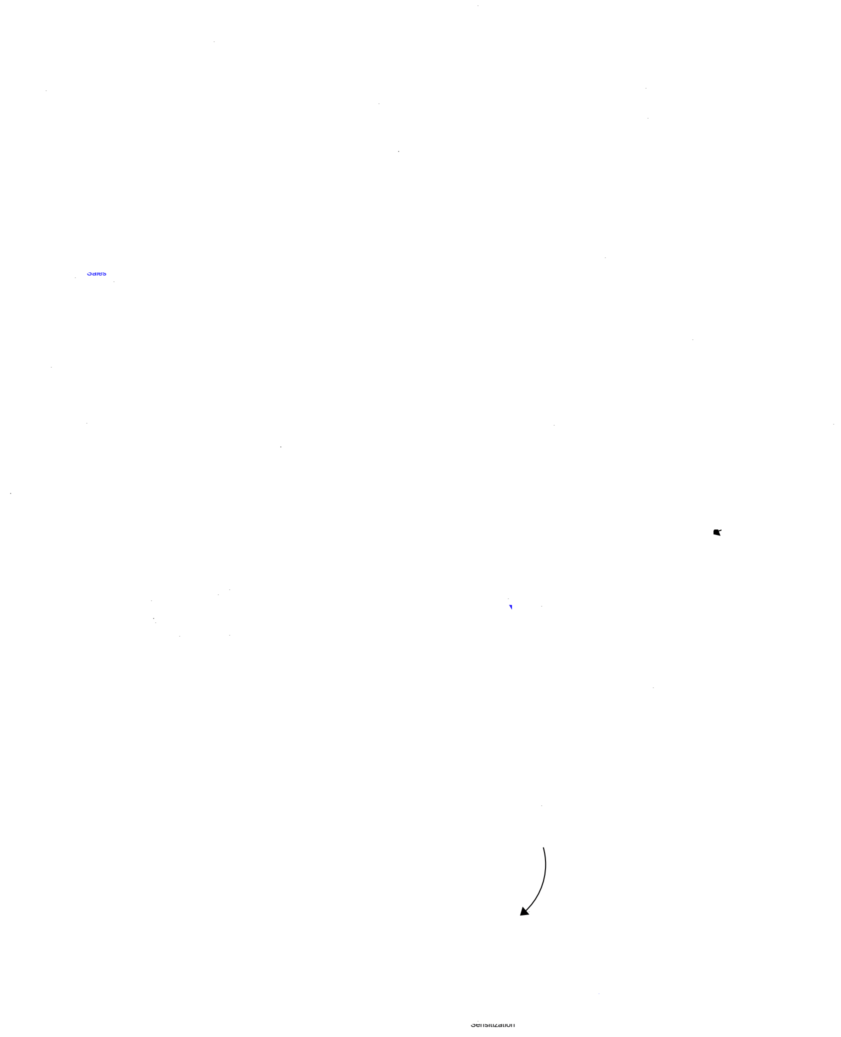

INTERVENTION TO REDUCE ANTIMICROBIAL USE ON FARM

1st rank - Governance of the value chain to reduce the use of antimicrobial use on the farm

2nd rank- The qualification of labour workforce on the farm-level

3rd rank - Improving the environment of the farm to reduce the incidence of disease infection

4th rank - Improving the biosecurity measures at the farm level to reduce disease infection

5th rank - Procurement of healthy day-old chicks to decrease disease infection
